# Supplementary material for: Tamarind Seed Coat: A Catechin-Rich Source with Anti-Oxidation, Anti-Melanogenesis, Anti-Adipogenesis and Anti-Microbial Activities
Source: Molecules. 2022 Aug 20;27(16):5319. doi: 10.3390/molecules27165319 (PMC9415986; doi:10.3390/molecules27165319)
Supplement: Supplementary file 1 [file molecules-27-05319-s001.zip › molecules-1864250-supplementary.pdf]

Tamarind Seed Coat: A Catechin-Rich Source with Anti-Oxidation, Anti-Melanogenesis, Anti-Adipogenesis and Anti-Microbial Activities

Supplementary Materials

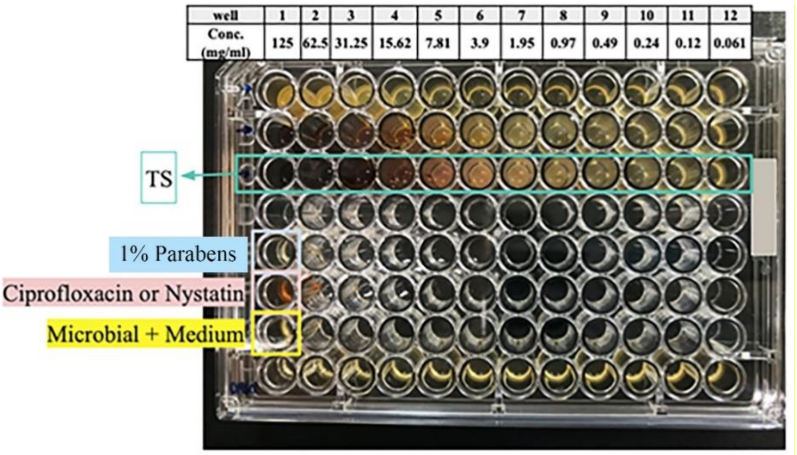

Figure S1.: Microdilution in 96-well plate assay.
